# Surfactant protein D attenuates sub-epithelial fibrosis in allergic airways disease through TGF-β

**DOI:** 10.1186/s12931-014-0143-9

**Published:** 2014-11-29

**Authors:** Hirohisa Ogawa, Julie G Ledford, Sambuddho Mukherjee, Yoshinori Aono, Yasuhiko Nishioka, James J Lee, Keisuke Izumi, John W Hollingsworth

**Affiliations:** Department of Cell Biology, Duke University Medical Center, Durham, North Carolina USA; Department of Medicine, Duke University Medical Center, Durham, North Carolina USA; Department of Respiratory Medicine and Rheumatology, Institute of Health Bioscience, University of Tokushima Graduate School, Tokushima, Japan; Department of Molecular and Environmental Pathology, Institute of Health Bioscience, University of Tokushima Graduate School, Tokushima, Japan; Department of Biochemistry and Molecular Biology, Division of Pulmonary Medicine, Mayo Clinic Arizona, Scottsdale, Arizona USA; Department of Medicine, Wexner Medical Center at Ohio State University, Columbus, Ohio USA; Davis Heart & Lung Research Institute at Ohio State University, 473 West 12th Avenue, Columbus, OH USA

**Keywords:** Surfactant protein D, Asthma, Fibrosis, Airway remodeling, Eosinophil, Transforming growth factor beta

## Abstract

**Background:**

Surfactant protein D (SP-D) can regulate both innate and adaptive immunity. Recently, SP-D has been shown to contribute to the pathogenesis of airway allergic inflammation and bleomycin-induced pulmonary fibrosis. However, in allergic airways disease, the role of SP-D in airway remodeling remains unknown. The objective of this study was to determine the contribution of functional SP-D in regulating sub-epithelial fibrosis in a mouse chronic house dust mite model of allergic airways disease.

**Methods:**

C57BL/6 wild-type (WT) and SP-D−/− mice (C57BL/6 background) were chronically challenged with house dust mite antigen (Dermatophagoides pteronyssinus, Dp). Studies with SP-D rescue and neutralization of TGF-β were conducted. Lung histopathology and the concentrations of collagen, growth factors, and cytokines present in the airspace and lung tissue were determined. Cultured eosinophils were stimulated by Dp in presence or absence of SP-D.

**Results:**

Dp-challenged SP-D−/− mice demonstrate increased sub-epithelial fibrosis, collagen production, eosinophil infiltration, TGF-β1, and IL-13 production, when compared to Dp-challenged WT mice. By immunohistology, we detected an increase in TGF-β1 and IL-13 positive eosinophils in SP-D−/− mice. Purified eosinophils stimulated with Dp produced TGF-β1 and IL-13, which was prevented by co-incubation with SP-D. Additionally, treatment of Dp challenged SP-D−/− mice with exogenous SP-D was able to rescue the phenotypes observed in SP-D−/− mice and neutralization of TGF-β1 reduced sub-epithelial fibrosis in Dp-challenged SP-D−/− mice.

**Conclusion:**

These data support a protective role for SP-D in the pathogenesis of sub-epithelial fibrosis in a mouse model of allergic inflammation through regulation of eosinophil-derived TGF-β.

**Electronic supplementary material:**

The online version of this article (doi:10.1186/s12931-014-0143-9) contains supplementary material, which is available to authorized users.

## Background

Surfactant is a lipoprotein complex that resides at the air-liquid interface of the lungs and is most commonly known for its role in reducing surface tension. Surfactant is produced by alveolar type II cells and airway Clara cells [1] and is composed of approximately 10% proteins, which includes surfactant protein (SP)-A, SP-B, SP-C and SP-D. SP-A and SP-D are members of collectin family of proteins and can modulate innate immunity. Previous reports have shown that SP-D can enhanced pulmonary clearance of pathogens including; *Pseudomonous aerginosa* [2], *Klebsiella pneumonia* [3], respiratory syncytial virus (RSV) [4] and Influenza virus [5]. Furthermore, SP-D has also been shown to modify allergic responses in the lungs and can bind to several common allergens, including house dust mite (*Dermatophagoides pteronyssinus*, Dp) [6], *Aspergillus fumigatus,* (Af) [7] and pollen granules [8]. Additionally SP-D reduce airway hyperresponsiveness (AHR) and eosinophilia in either ovalbumin (OVA) [9] or in Af [10] murine models of allergic airways disease and SP-D administration after antigen challenge can attenuate eosinophila and Th2 cytokine production in Dp-sensitized mice [11-13]. While SP-D can attenuate AHR and eosinophilia in these allergic models, the role of SP-D in remodeling of the airways remains unexplored.

Airway remodeling is central to the pathogenesis of asthma and can include sub-epithelial fibrosis, mucus cell hyperplasia and smooth muscle hypertrophy/hyperplasia. A better understanding of the factors that regulate the pathogenesis of sub-epithelial fibrosis may provide an opportunity for novel interventions in chronic bronchial asthma. Previous work demonstrated that both SP-A and SP-D can mitigate pulmonary fibrosis in mouse models of lung injury. For example, SP-A-deficient and SP-D-deficient mice are susceptible to bleomycin-induced lung injury and display increased cellular inflammation, more severe lung fibrosis, and reduced survival [14,15]. Studies using the bleomycin lung fibrosis model support that SP-D attenuate pulmonary fibrosis through both regulation of TGF-β1 and PDGF-AA production, as well as, limiting fibrocyte migration into the lung [16]. Clinical relevance of these findings is supported by the association between serum levels of either SP-A or SP-D and mortality in patients with pulmonary fibrosis [17,18].

Based on these previous observations, we used a model of chronic exposure to Dp to test the hypothesis that SP-D would attenuate the development of sub-epithelial fibrosis in an allergic airways disease. Present findings here suggest that SP-D plays a protective role in allergic airways by reducing the development of sub-epithelial fibrosis.

## Materials and methods

Detailed methods are described in the supporting information.

### Preparation of antigen

House-dust mite antigen (Dermatophagoides pteronyssinus, Dp) was purchased from Cosmobio Ltd (Tokyo, Japan). Endotoxin levels were reduced using endotoxin removal solution (Sigma-Aldrich, Japan) to <0.02 EU/mg.

### Animal protocol

All mouse studies were carried out in strict accordance with the recommendations in the Guide for the Care and Use of Laboratory Animals of the National Institutes of Health. The protocol was approved by the Institute of Animal Care and Use Committee (IACUC) at Duke University. All surgery was performed under Ketamine (50 mg/kg)/Xylazine (5 mg/kg) anesthesia and all efforts were made to minimize suffering.

**Figure 1:**
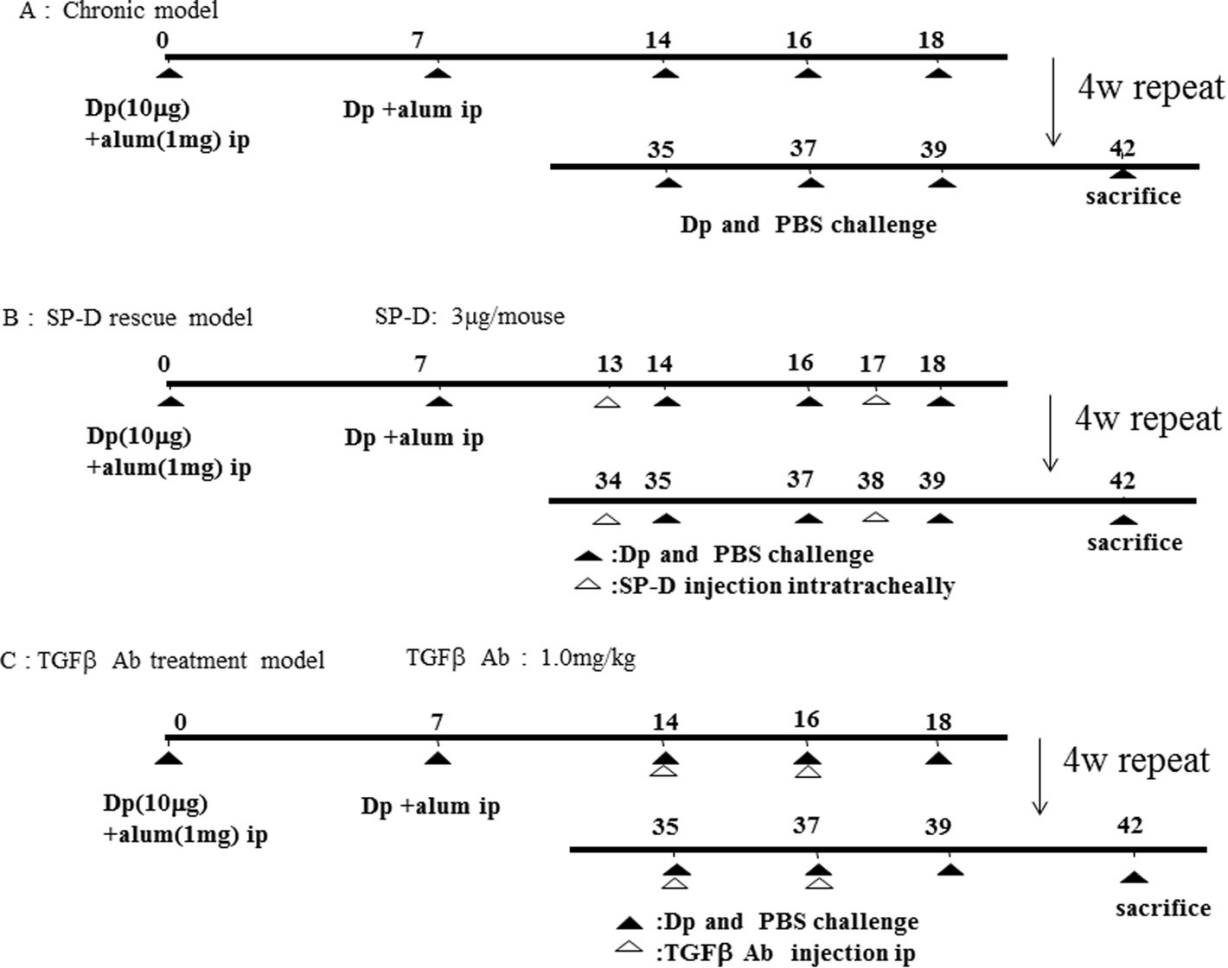
**Experimental mouse protocols. (A)** Model of sensitization and chronic challenge to Dp **(B)** SP-D rescue model **(C)** Anti-TGF-β1 antibody treatment model.

SP-D knockout (SP-D−/−) mice (C57BL/6 background) and IL-5 transgenic mice (C57BL/6 background) were generated as previously described [19,20]. Wild-type (WT) C57BL/6 mice were purchased from The Jackson Laboratory and bred in-house to control for environmental conditions. 6–10 week old mice were sensitized and challenged by Dp as described previously [21] (Figure 1). 3–5 mice per group were used in each experiment and these experiments were repeated for 2–3 times. Data from experiments were pooled for analysis. Bronchoalveolar lavage (BAL) was performed and lungs were harvested for histopathology and lung homogenization [21].

### Exogenous SP-D administration *in vivo*

Recombinant SP-D was isolated from Chinese hamster ovary cells expressing rat SP-D protein as described previously [22]. Recombinant SP-D (3 μg in 50 μl PBS) or 50 μl PBS as control was administered into Dp-challenged SP-D−/− mouse by oropharyngeal aspiration as described previously [15,16] twice weekly from days 13 to 38 (Figure 1).

### Anti-TGF-β1 antibody administration *in vivo*

1.0 mg/kg of Anti-TGF-β1 antibody (R&D Systems, Minneapolis, MN) or 1.0 mg/kg of IgG isotype antibody(R&D Systems) as control was administered intraperitoneally into Dp challenged WT and SP-D−/− mouse twice weekly from days 14 to 37 (Figure 1).

### Eosinophil purification and *in vitro* experiment

Eosinophils were purified from blood of IL-5 transgenic mouse as described previously and purity was determined to be greater than 95% [23]. Eosinophils (4x10^5^) were incubated in 48 well plates in the presence or absence of SP-D for 1 hr. After pre-incubation, eosinophils were stimulated by various concentration of Dp solution for 24 hrs. SP-D was boiled by 100°C for 10 min and was used as heat-inactivated SP-D [24].

### Histology

Lung tissue was fixed in 10% formalin and embedded in paraffin. Three-micrometer thick sequential sections were performed. Sections for fibrosis were stained with Gomori’s trichrome stain. Sequential sections were stained with Luna-modified stain and TGF-β1 and IL-13 immunohistochemistry (IHC). Both primary antibodies were purchased from Abcam (Cambridge, UK). IHC were performed as described previously [25]. Morphological analysis was performed quantitatively by Image J (National Institutes of Health).

### Measurements of total protein and cytokine concentrations

Harvested lungs were homogenized in lysis buffer (Cell Signaling Technology, Inc. Danvers, MA) containing 1 mM phenylmethanesulfonyl fluoride (PMSF, Sigma-Aldrich) using Savant FastPrep FP120 Homogenizer (Thermo Scientific, Waltham, MA). Protein concentrations were determined by the BCA method (Pierce, Rockford, IL). Cytokines/growth factor were measured with commercial ELISA kits (details were described in Additional file 1). The values graphed for cytokine were adjusted to the total protein concentration of the respective lung samples.

### Collagen assay

Lungs were homogenized in 0.5 M acetic acid (50 volumes to wet lung weight) containing about 1 mg/ml pepsin (Sigma) using Savant FastPrep FP120 Homogenizer (Thermo Scientific, Waltham, MA). Total lung collagen was determined using the Sircol Collagen Assay kit (Biocolor Ltd., Belfast, Northern Ireland) according to the manufacturer’s instructions. The values graphed for collagen were adjusted to the total protein concentration of the respective lung samples.

### Flow cytometry

The lungs were minced and enzymatically digested (DNAse and Collagenase) for 1 hr at 37°C. Cells were stained by various fluorescence conjugated -antibodies (details were described in Additional file 1). The stained cells were analyzed by FACS using a BD LSRII and BD FACS Canto II (San Diego, CA) for acquisition.

### Statistical analysis

Comparisons between groups were analyzed using one-way ANOVA with post-hoc Tukeys analysis. Some comparisons between groups made with Student T-test without ANOVA (GraphPad Prism, version 5.0; GraphPad Software, Inc., San Diego, CA). Data are presented as mean ± SEM. Differences were considered statistically significant if p values were less than 0.05.

## Results

### Sub-epithelial airway fibrosis in Dp challenged mice

**Figure 2:**
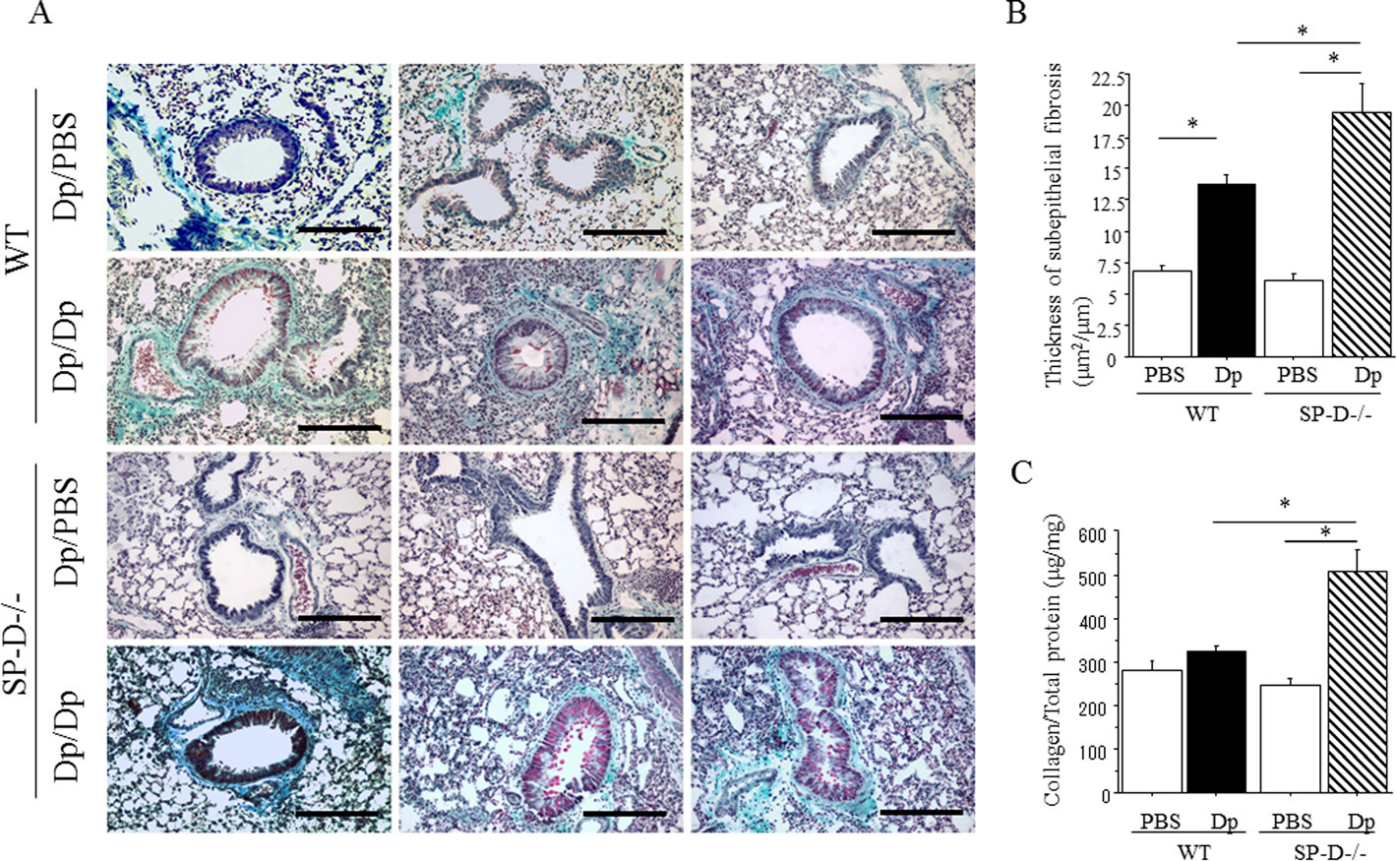
**Sub-epithelial airway fibrosis in SP-D−/− mice after Dp chronic exposure. (A)** Representative photomicrographs of lungs from either wild-type (WT) C57BL/6 mice or SP-D−/− mice stained with Gomori’s trichrome on day 42 after PBS or Dp challenge. Vertical rows were arranged by group and horizontal rows were results representative of 3 independent experiments. Magnification: 100x. Scale bar = 200 μm. Dp/PBS: Dp sensitized and PBS challenged mice. Dp/Dp: Dp sensitized and Dp challenged mice. **(B)** Morphological analysis of sub-epithelial fibrosis. **(C)** Collagen content in the lungs of SP-D−/− mice or C57BL/6 (WT) mice on day 42 after saline or Dp challenge. Subepithelial fibrosis thickness and collagen production was measured as described in Material and Methods. Data are presented as means ± SEM obtained from 3 different experiments. 3–5 mice per group were used in each experiment (N = 10-15). White bars: PBS-challenged mice in each group, Black bar: Dp-challenged WT mice. Shaded bar: Dp-challenged SP-D−/− mice. *p < 0.05.

Chronic Dp exposure increased sub-epithelial fibrosis in Dp-challenged WT and SP-D−/− mice compared to PBS challenged mice (Figure 2A). When compared with Dp-challenged WT mice, Dp-challenged SP-D−/− mice demonstrate markedly increased sub-epithelial fibrosis after chronic exposure (Figure 2A). The thickness of sub-epithelial fibrosis was increased in all Dp-challenged groups when compared to PBS challenged groups. However, the thickness of sub-epithelial fibrosis of Dp challenged SP-D−/− mice was significantly greater than that of Dp-challenged WT mice (Figure 2B). Likewise, the amount of collagen in the lungs of Dp-challenged SP-D−/− mice was significantly increased compared to the lungs of Dp-challenged WT mice and PBS-challenged SP-D−/− mice (Figure 2C).

### Cellular inflammation in Dp-challenged mice

**Figure 3:**
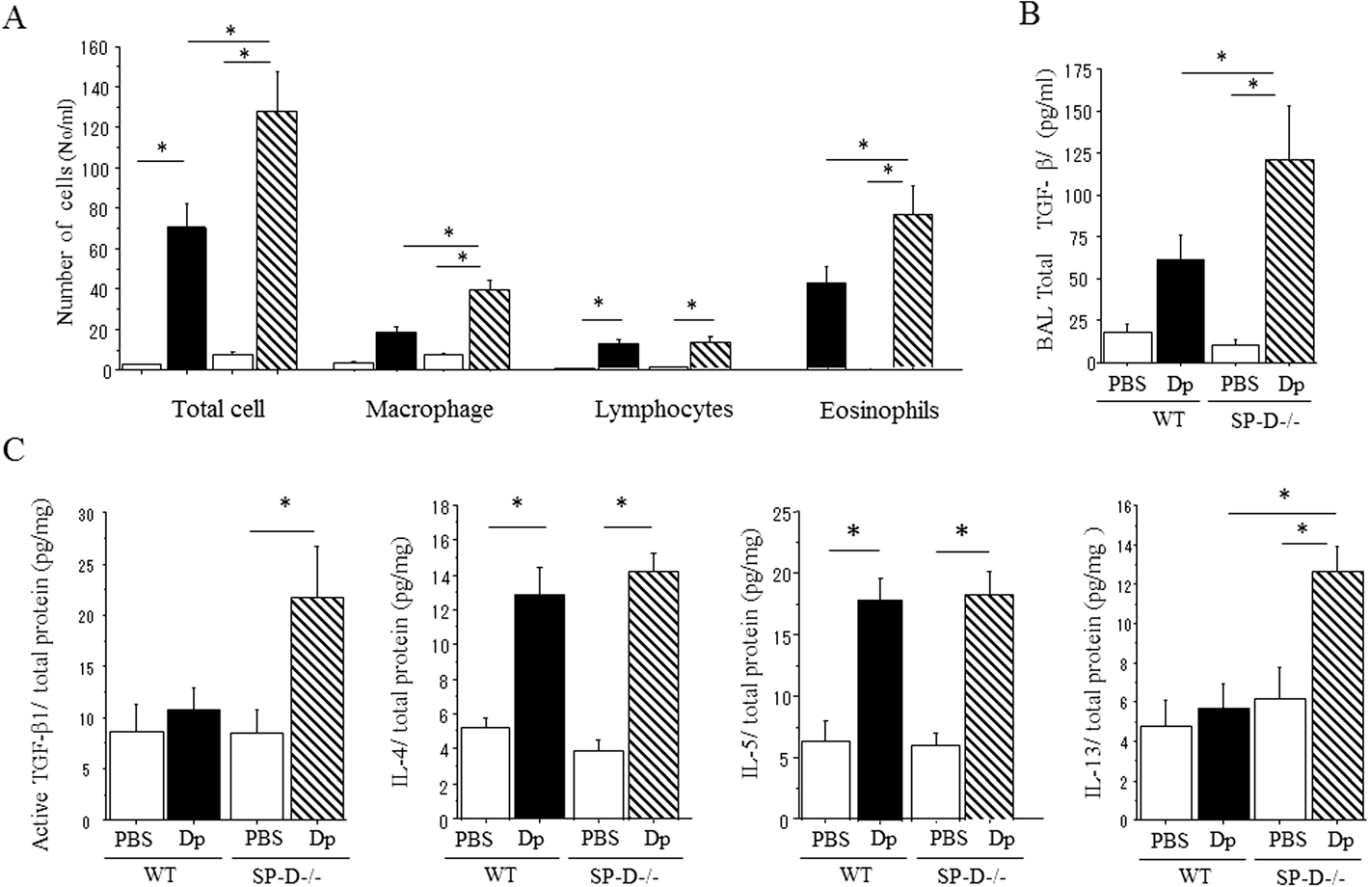
**BAL fluid cell analysis and cytokine concentration in Dp-challenged SP-D−/− mice. (A)** Bronchoalveolar lavage (BAL) was performed for total cell count and differentials. **(B)**; Level of TGF-β1 in BAL fluid. **(C)** Level of TGF-β1 and Th2 cytokines in lung homogenate. Cytokines in BAL and lung homogenates was measured by ELISA. Data are presented as means ± SEM obtained from 3 different experiments. 3–5 mice per group were used in each experiment (N = 9-14). White bars: PBS challenged mice in each groups. Black bar: Dp challenged WT mice. Shaded bar: Dp challenged SP-D−/− mice. *p < 0.05.

Differential cell counts from the bronchial alveolar lavage fluid (BALF) and cytokine/growth factor concentrations in BALF and whole lungs were examined to evaluate the allergic inflammation of DP-challenged mice. Although there were no observed differences in the number of lymphocytes in BALF among both groups of Dp-challenged mice, the total cells, eosinophils, and macrophages in BALF were significantly increased in Dp-challenged SP-D−/− mice when compared to Dp-challenged WT mice, (Figure 3A).

TGF-β1 is recognized to be a key cytokine driving fibrotic lung disease [16]. Total TGF-β1 concentration in BALF of Dp challenged SP-D−/− mice was significantly increased when compared to Dp-challenged WT mice and PBS challenged SPD−/− mice (Figure 3B). Active TGF-β1 of lung homogenate in Dp challenged SP-D−/− mice tended to increase when compared to Dp-challenged WT mice although there are no statistically significant differences observed (Figure 3C). These findings suggest that functional TGF-β1 was produced around inflammatory site of lung in SP-D deficient mice.

Several Th2 cytokines, IL-4, IL-5 and IL-13, were undetectable in BALF, but were present in the lung homogenates. While there were no detectable differences in IL-4 and IL-5 between both groups of Dp-challenged mice, SP-D−/− mice had significantly increased IL-13 production when compared to WT after Dp-challenge (Figure 3C).

### Th2/Th1 cell population and cytokines in Dp-challenged mice

**Figure 4:**
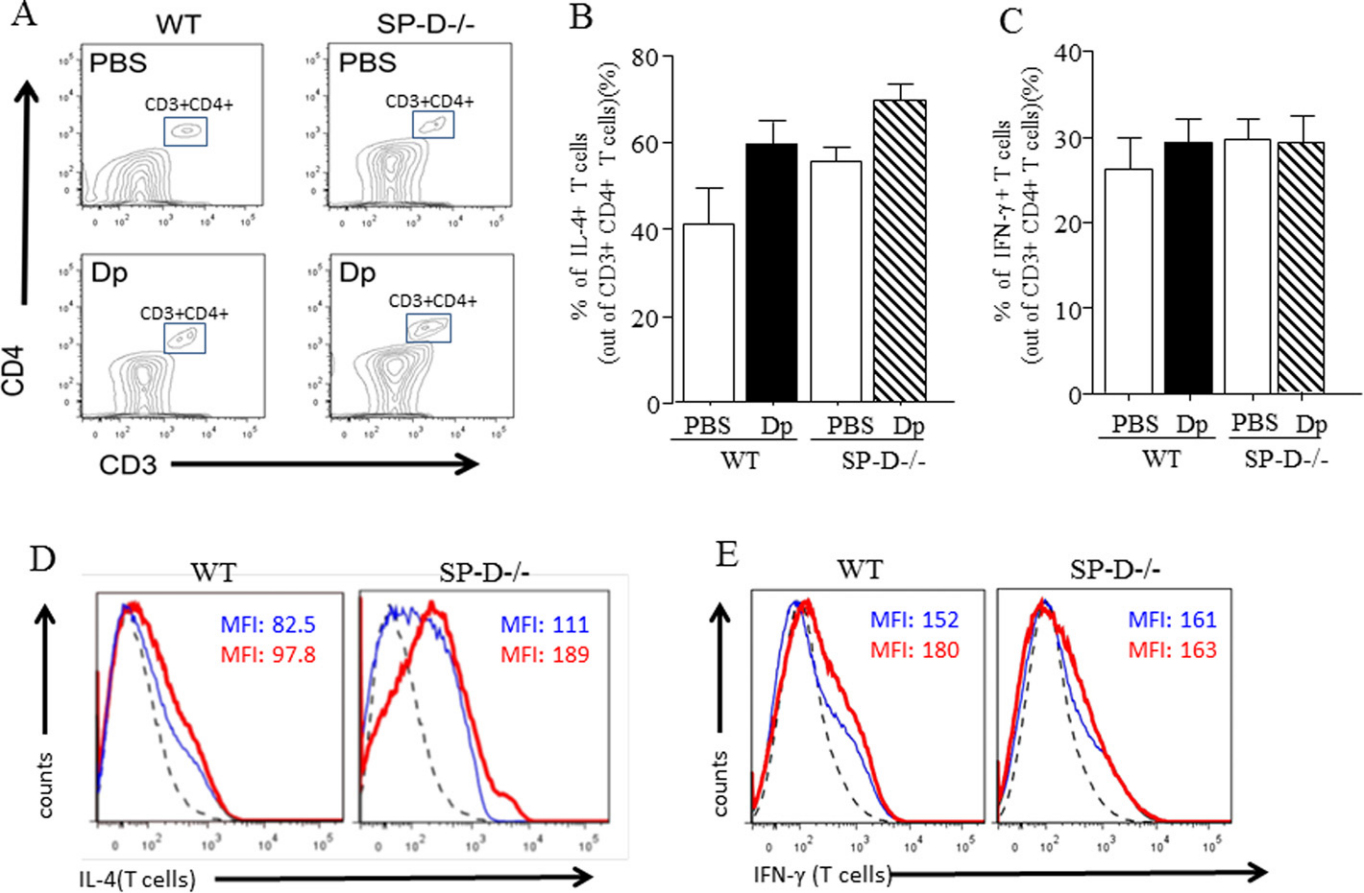
**Th2/Th1 cell population in Dp challenged mice.** Th2/Th1cell population were examined by flow cytometry. **(A)**: Gating information and contour plots of CD3+ CD4+ lymphocytes from lung digests, among the 4 mice groups. Lymphocytes were initially examined for dual expression of CD3 and CD4. Data was representative in 3 different experiments, with n = 3 to 5 mice per condition. **(B)**: IL-4 positive Th cells population. **(C)**: IFN-γ positive Th cells population. IL-4/IFN-γ positive cells were analyzed in CD3 + CD4 T cells. Percent of intracellular cytokine positive cells relative to CD3 + CD4+ T cells of lung digests were analyzed by flow cytometry. Data were representative across 3 independent experiments and were presented as means ± SEM of 3–5 mice per group. White bars: PBS challenged mice in each groups. Black bar: Dp challenged WT mice. Shaded bar: Dp challenged SP-D−/− mice. **(D)**: mean fluorescence intensity (MFI) of IL-4 per T cell basis. **(E)**: MFI of IFN-γ per cell basis. per cell basis. Data was representative in 3 different experiments, with n = 3 to 5 mice per condition. Stippled line: negative control IgG. Blue solid line: PBS challenged mice. Red bold line: Dp challenged line. MFI: mean fluorescence intensity.

To determine whether Th lymphocytes affected IL-13 production in SP-D−/− mice, we examined the intracellular cytokine profile of Th2/Th1 cells that were present in the total CD4+ cell population (CD3+/CD4+) from the homogenized and digested lung tissue by flow cytometry (Figure 4A). Although, the percentage of IL-4+ T cells trended towards an increase by Dp exposure when compared with PBS challenged mice, there were no detectible differences in percentage of IL-4+ T cells between either group of Dp-challenged mice (Figure 4B). Additionally, there were no detectible differences in the number of IFN-γ + T cells from lung tissue between all groups of mice (Figure 4C). To determine Th2/Th1 cytokine production per T cells basis, we analyzed mean fluorescence intensity (MFI) of IL-4 and IFN-γ per cell basis. MFI of IL-4 in Dp challenged SPD−/− mice was only slightly increased compared to PBS challenged SPD−/− mice, and appears to be within 2 fold increase compared to Dp challenged WT mice (Figure 4D). There were no detectable differences in MFI of IFN-γ between all groups of mice (Figure 4E). Based on these results, we did not observe detectible differences in Th2/Th1 lymphocyte populations and Th2/Th1 cytokines in SP-D−/− mice, which suggests that Th2/Th1 lymphocytes may not be a major contributor to the SP-D-dependent IL-13 production.

### Treatment with exogenous SP-D in Dp-challenged mice

**Figure 5:**
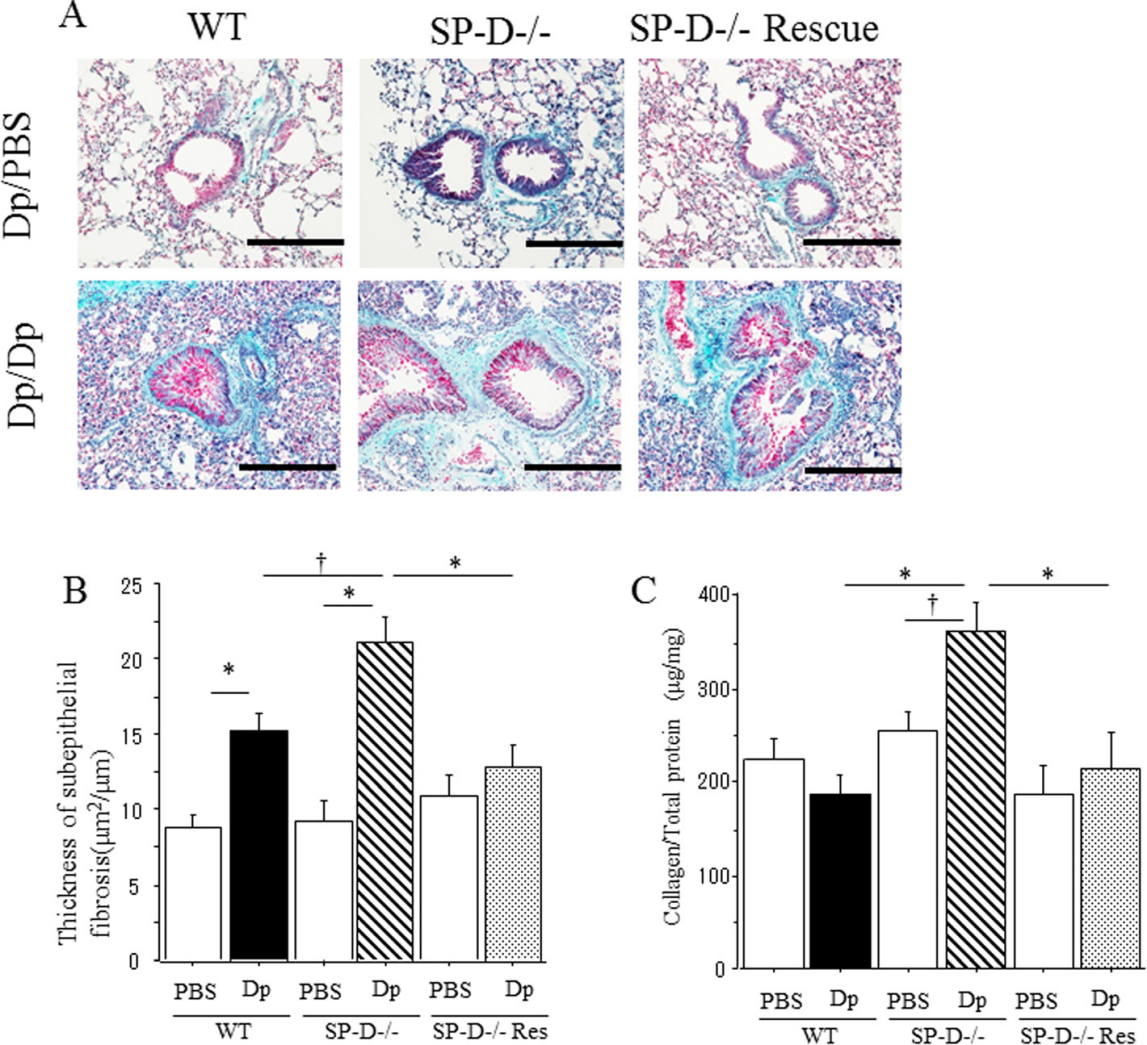
**Sub-epithelial fibrosis in Dp-challenged mice with treatment of exogenous SP-D. (A)** Representative photomicrographs of lungs from C57BL/6 WT, SP-D−/−, and SP-D−/− with SP-D rescue treatment were stained with Gomori’s trichrome on day 42 after PBS or Dp-challenge. Magnification: 100x. Scale bar =200 μm. Dp/PBS: Dp-sensitized and PBS-challenged mice. Dp/Dp: Dp-sensitized and Dp-challenged mice. **(B)** Morphological analysis of sub-epithelial fibrosis. Sub-epithelial fibrosis thickness was measured as described in Material and Methods. **(C)** Collagen production in lungs of C57/B6 (WT), SP-D−/−, and SP-D−/− with SP-D rescue treatment and on day 42 after saline or Dp challenge. Collagen was determined by Sircol collagen assay. In these bar graphs (B and C), data are presented as means ± SEM obtained from 3 different experiments. 3–5 mice per group were used in each experiment (N = 6-13). White bars: PBS challenged mice in each groups. Black bar: Dp challenged WT mice. Shaded bar: Dp challenged SP-D−/− mice. Dotted bars: Dp challenged SP-D−/− mice with SP-D rescue. *p < 0.05 ANOVA with post-hoc Tukeys; †p < 0.05 Student T-test.

**Figure 6:**
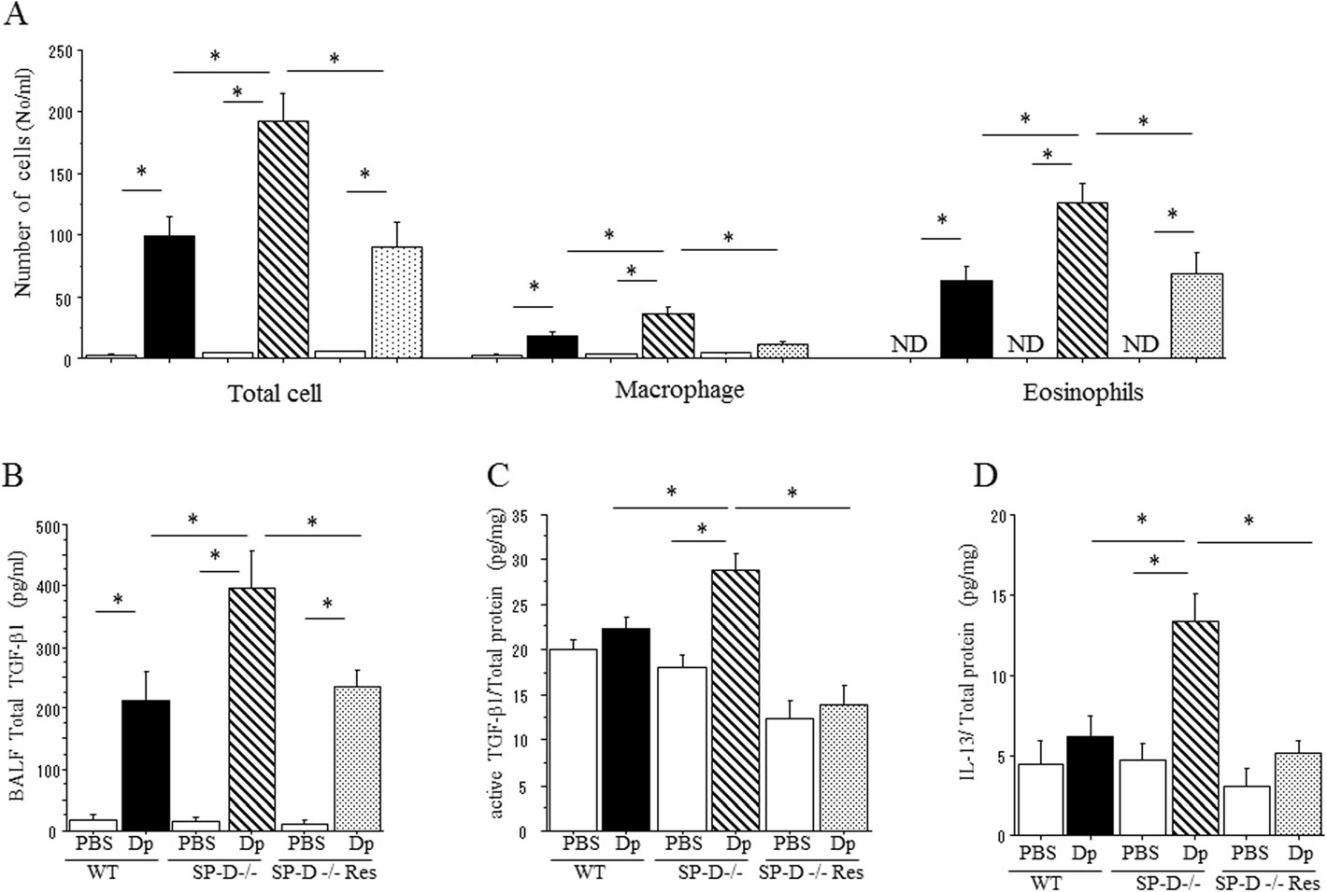
**BAL fluid cell analysis and cytokine concentration in Dp-challenged mice with treatment of SP-D. (A)** BAL was performed for total cell count, macrophages and eosinophils. **(B)**; Level of total TGF-β1 in BAL fluid. **(C)**; level of active TGF-β1 in lung homogenate. **(D)**; level of IL-13 in lung homogenate. Cytokines in BAL and lung homogenates was measured by ELISA. Data are presented as means ± SEM obtained from 3 different experiments. 3–5 mice per group were used in each experiment (N = 7-13). White bars: PBS challenged mice in each groups. Black bar: Dp challenged WT mice. Shaded bar: Dp challenged SP-D−/− mice. *p < 0.05.

To determine whether loss of SP-D directly affected the increase of sub-epithelial fibrosis, exogenous SP-D was administrated oropharyngeally into SP-D−/− mice (SP-D rescue treatment mice, SP-D−/− Res). Again, we observed that sub-epithelial fibrosis in SP-D−/− mice was increased by chronic Dp exposure as compared to WT Dp exposed mice. However, in Dp-challenged SP-D−/− mice given the SP-D rescue treatment, sub-epithelial fibrosis was attenuated (Figure 5A). Similarly, the thickness of sub-epithelial fibrosis surrounding the bronchus was significantly reduced in Dp-challenged SP-D−/− mice given SP-D rescue treatment when compared to Dp-challenged SP-D−/− mice given vehicle control (Figure 5B). The concentration of collagen in the lungs of Dp challenged SP-D−/− mice was significantly increased compared to that of Dp-challenged WT mice. SP-D rescue treatment also significantly decreased collagen concentration in lungs of Dp-challenge SP-D−/− mice (Figure 5C). Moreover, SP-D rescue treatment also significantly decreased the cellular inflammation (Figure 6A) and total TGF-β1 of BALF (Figure 6B) of Dp-challenged SP-D−/− mice as compared to vehicle treated controls. We observed similar patterns that the increases of both active TGF-β1 and IL-13 in SP-D−/− were reduced by SP-D rescue treatment (Figure 6C and D).

### TGF-β1 and IL-13 positive eosinophil infiltration in lungs of Dp-challenged mice

**Figure 7:**
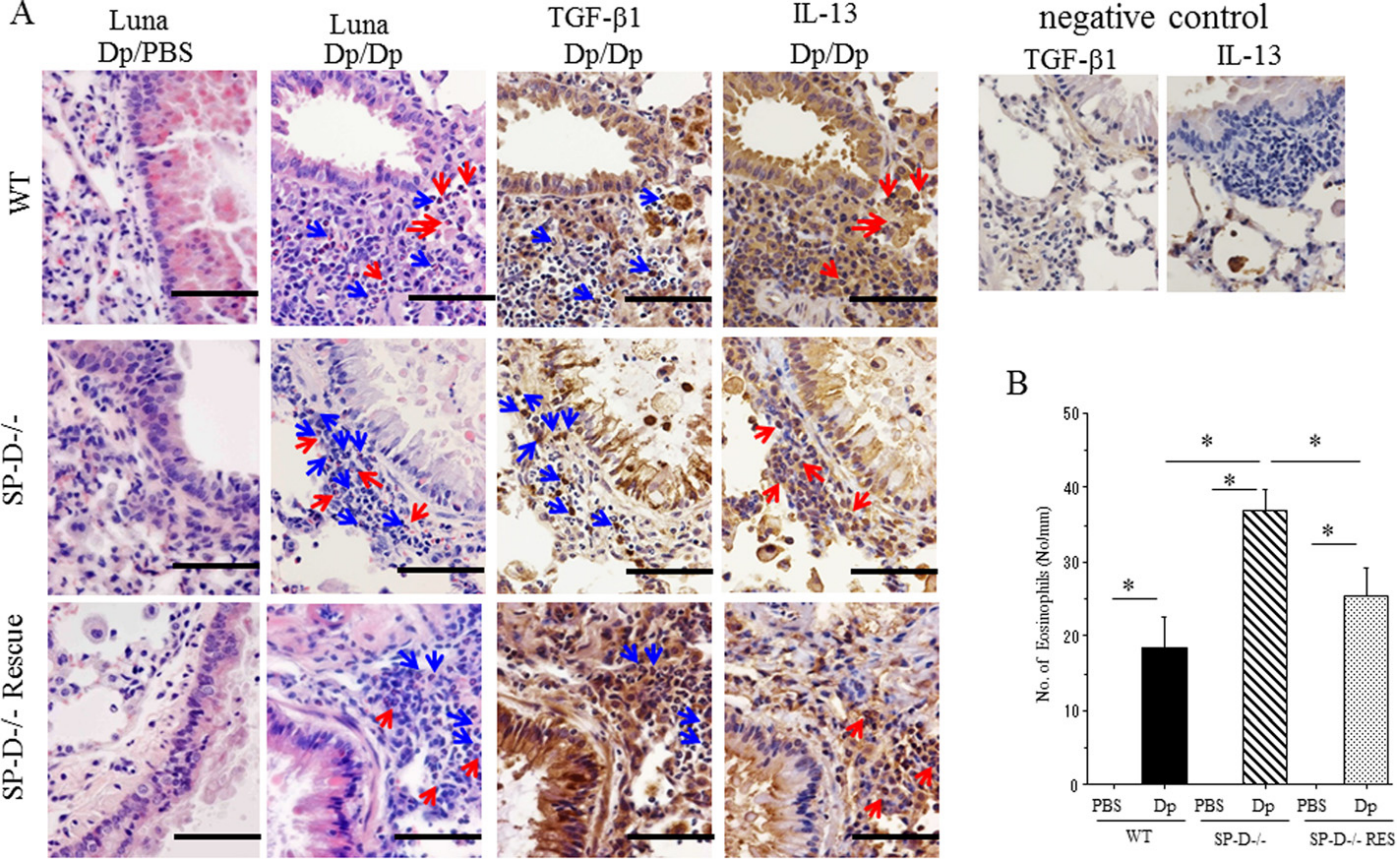
**Eosinophil infiltration and TGF-β1 and IL-13 production in lungs of Dp-challenged mice. (A)** Photomicrographs of Luna staining (eosinophils) and immunohistochemistry for TGF-β1 and IL-13. Photographs were representative in 3 different experiments. Magnification: 400x. Scale bar =50 μm. Sequential staining were performed between Luna staining and immunohistochemistry for TGF-β1and IL-13. Blue arrows: TGF-β1 positive eosinophils; Red arrows: IL-13 positive eosinophils. Dp/PBS: Dp-sensitized and PBS-challenged mice. Dp/Dp: Dp-sensitized and Dp-challenged mice. **(B)** Morphological analysis of eosinophil infiltration in lungs Eosinophils under the sub-epithelial region were counted as described in Material and Methods. Data are presented as mean ± SEM obtained from 3 different experiments. 3–5 mice per group were used in each experiment (N = 7-11). White bars: PBS challenged mice in each groups. Black bar: Dp challenged WT mice. Shaded bar: Dp challenged SP-D−/− mice. Dotted bars: Dp challenged SP-D−/− mice with SP-D rescue. *p < 0.05.

Previous reports have shown that allergic inflammation, including eosinophilia, is related to the establishment of airway remodeling [26,27]. In our studies, histological examination with Luna modified stain demonstrated that peribronchiolar eosinophils (red in cytoplasm) were present in all groups of Dp-challenge mice when compared to PBS-challenged mice (Figure 7A). However, the number of tissue eosinophils was increased in Dp-challenged SP-D−/− mice when compared to Dp-challenged WT mice (Figure 7B). SP-D rescue treatment also significantly decreased the eosinophil infiltration into the tissue of Dp-challenged SP-D−/− mice (Figure 7B). In order to determine if eosinophils are the source of TGF-β1 and IL-13 in Dp-challenged SP-D−/− mice, we performed Luna-modified stain and immunohistochemistry using sequential staining techniques. Using this techniques, we are able to show that many of the infiltrated eosinophils which are positive for Luna-modified stain, are also positive for TGF-β1 (blue arrows) and for IL-13 (red arrows) (Figure 7A). As shown in Table 1, while the percentage of TGF-β1 positive and IL-13 positive eosinophils was similar among the groups, the total number of TGF-β1 positive and IL-13 positive eosinophils was significantly increased in SPD−/− mice compared to WT and SP-D−/− rescue mice. Interestingly, bronchial epithelial cells were also positive for TGF-β1 (Figure 7A). Since the percentage of TGF-β1 positive epithelial cells was not increased in SP-D−/− mice (Table 1), this suggests that epithelium derived TGF-β1 may not be affected by SP-D in this model. These findings further support the notion that eosinophils are an important source of both TGF-β1 and IL-13 and may be regulated by SP-D.

**Table 1:** **Histological data of eosinophils and epithelial cells by IHC**

		**TGF + Eo**	**% of TGF + Eo**	**IL13 + Eo**	**% of IL13 + Eo**	**% of TGF + epithelial cells**
		**(No./mm)**	**(%)**	**(No./mm)**	**(%)**	**(%)**
WT	Dp/Dp	7.67 ± 1.90	42.34 ± 3.71	8.41 ± 1.79	41.83 ± 3.87	88.35 ± 5.14
SPD−/−	Dp/Dp	14.51 ± 1.92^*^	41.91 ± 3.90	15.32 ± 2.59^*^	41.58 ± 4.31	91.65 ± 3.68
SPD−/− rescue	Dp/Dp	9.06 ± 1.45^†^	40.38 ± 4.29	8.65 ± 1.32^†^	37.34 ± 2.43	97.07 ± 1.31

TGF-β1 and IL-13 were stained by immunohistochemistry (IHC). Methods to evaluate the IHC stain were described in Material and Methods. Data are presented as means ± SEM obtained from 3 different experiments. 3–5 mice per group were used in each experiment (N = 7-11). Eo: eosinophils. Dp/Dp: Dp-sensitized and Dp-challenged mice). *: P < 0.05 compared with Wt mice. ^†^: P < 0.05 compared with SPD−/− mice.

### SP-D regulates eosinophil-derived TGF-β1 and IL-13

**Figure 8:**
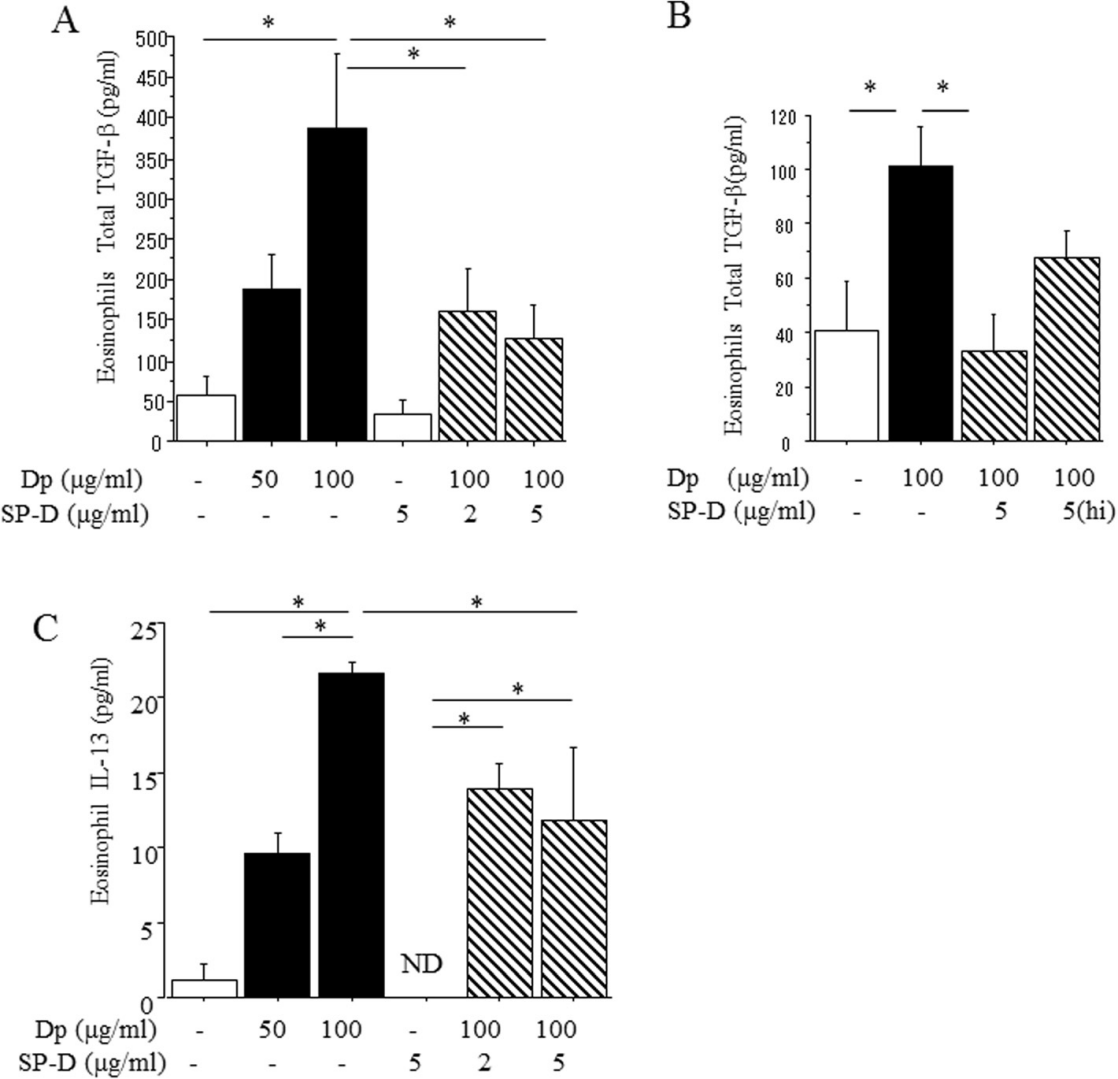
**SP-D regulates eosinophil-derived TGF-β1 and IL-13.** Effect of SP-D on eosinophils-derived TGF-β1 and IL-13 production was examined in vitro experiment. **(A)**: Level of total TGF-β1 in eosinophil culture supernatant. **(B)**: Level of total TGF-β1 in supernatant of eosinophil cultures in the presence of normal and heat inactivated SP-D. **(C)**: Level of IL-13 in eosinophil culture supernatant. Eosinophils from IL-5 transgenic mice were incubated *in vitro* with Dp for 24 hr in the presence or absence of SP-D pre-incubation as described Material and Methods. TGF-β1 and IL-13 in culture supernatants was determined by ELISA. Data are presented as means ± SEM obtained from 2 different experiments (N = 6). White bars: control. Black bars: Dp stimulation at different concentrations. Shaded bars: Dp stimulation with SP-D pre-incubation at different concentrations. *p < 0.05.

To determine if SP-D directly regulates eosinophil function, *in vitro* experiments were performed with purified eosinophils that were stimulated with Dp in the presence or absence of SP-D. Dp-stimulation significantly increased TGF-β1 production from eosinophils in a dose dependent manner (Figure 8A). Interestingly, SP-D pre-treatment of eosinophils significantly reduced TGF-β1 production at both 2 μg/ml and 5 μg/ml doses (Figure 8A). Heat-inactivated SP-D is less effective in inhibiting TGF-β production from eosinophils when compared to multimeric SP-D (Figure 8B). In addition, IL-13 production by isolated eosinophils was also significantly increased by Dp stimulation in a dose dependent manner, which was significantly reduced by SP-D co-incubation (Figure 8B). Taken together, these results demonstrate that SP-D can directly attenuate Dp-induced eosinophil-derived TGF-β1 and IL-13 production.

### TGF-β blockade inhibits sub-epithelial fibrosis in the SP-D deficient animal

**Figure 9:**
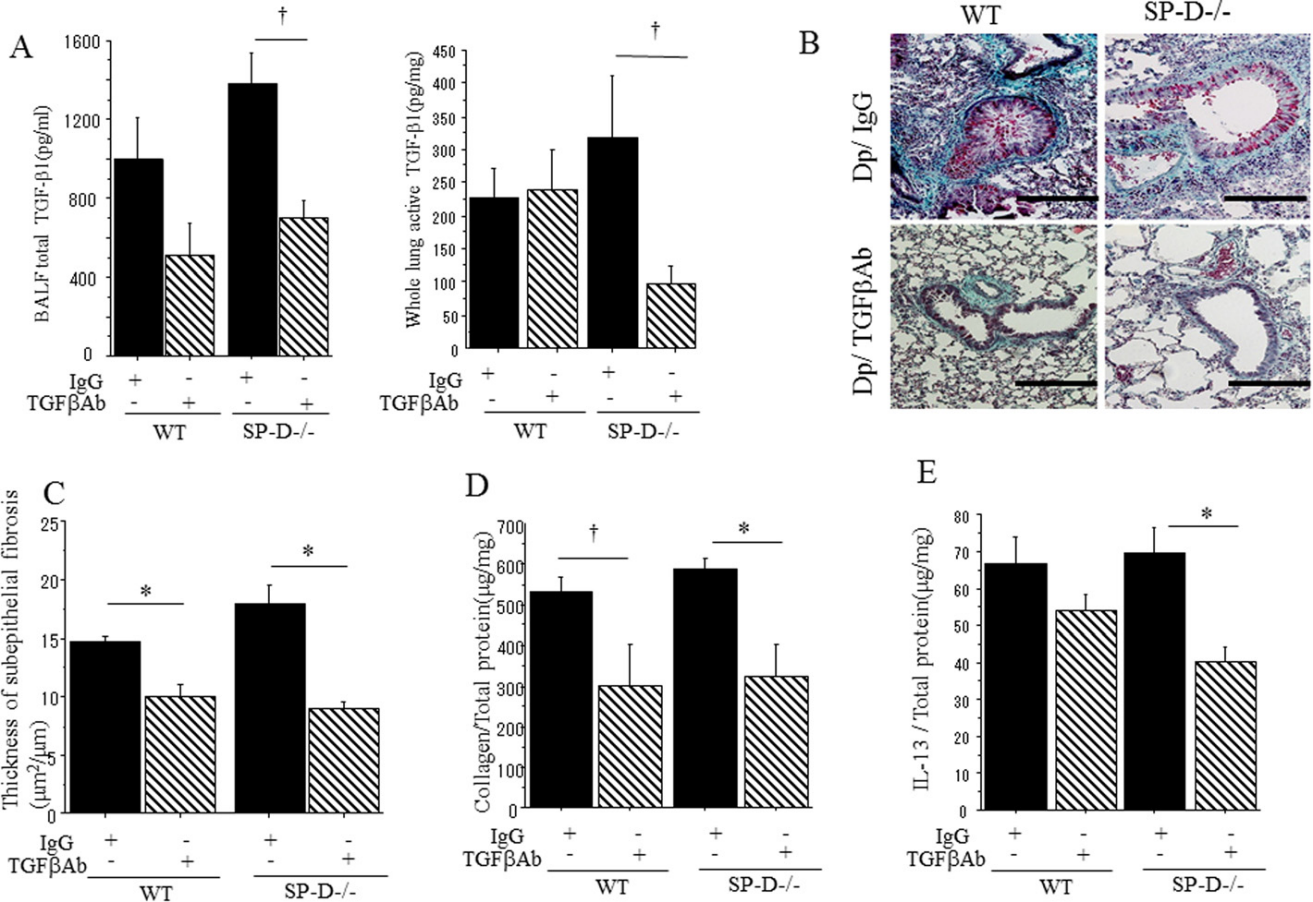
**Anti-TGF-β1 antibody treatment of Dp-challenged C57BL/6 and SP-D−/− mice.** SP-D−/− mice were treated with an anti TGF-β1 antibody and IgG isotype control to examine whether TGF-β1 was important for sub-epithelial fibrosis. **(A)**: Total TGF-β1 in BALF and active TGF-β1 in lung homogenates. **(B)**: Photomicrographs of lungs stained with Gomori’s trichrome. They were representative of 3 different experiments. Magnification: 100x. Scale bar =200 μm. Dp/IgG: Dp sensitized and Dp challenged mice with IgG treatment. Dp/TGFβAb: Dp sensitized and Dp challenged mice with TGF-β antibody treatment. **(C)**: Subepithelial fibrosis thickness. **(D)**: Collagen production in lungs determined by Sircol collagen assay. (E): Level of IL-13 in lungs determined by ELISA. In these bar graphs **(A**,**C**,**D** and **E)**, data are presented as mean ± SEM obtained from 2 different experiments. 3–5 mice per group were used in each experiment (N = 6-8). Black bars: Dp challenged WT and SP-D−/− mice treated with IgG isotype control. Shaded bars: Dp challenged WT and SP-D−/− mice treated with an anti-TGF-β1 antibody. *p < 0.05 ANOVA with post-hoc Tukeys; †p < 0.05 Student T-test.

In order to determine whether TGF-β1 affected sub-epithelial fibrosis in the SP-D−/− mice, we blocked TGF-β1 in our Dp model. Treatment with TGF-β blocking antibody significantly reduced total and active TGF-β1 concentrations as measured in the BALF and whole lung homogenate (Figure 9A). TGF-β1 blockade reduced sub-epithelial fibrosis in both strain of Dp-challenge mice compared to Dp-challenge mice given the IgG isotype control administration (Figure 9B). Additionally, the thickness of sub-epithelial fibrosis in Dp challenged SP-D−/− mice with TGF-β1 Ab treatment was significantly reduced compared to Dp-challenged SP-D−/− mice given the IgG isotype control treatment (Figure 9C). The inhibitory effect of anti-TGF-β treatment in SPD−/− mice was higher than that in WT mice (WT vs SP-D−/−; 31.7% reduced *vs* 50.0% reduced, respectively) (Figure 9C). Collagen concentrations were also significantly decreased in SP-D−/− Dp challenged lungs that had been given TGF-β1 Ab treatment when compared to those given IgG isotype control treatment (Figure 9D). Interestingly, TGF-β1 Ab treatment significantly decreased IL-13 concentration in SP-D−/− mice, although it was not effective for WT mice (Figure 9E). These results support that TGF-β1 is key cytokine in establishing Dp-induced sub-epithelial fibrosis in mice that lack functional SP-D.

## Discussion

Sub-epithelial fibrosis is a major complication of chronic allergic airways disease and can result in fixed air-flow obstruction. Current understanding of the fundamental molecular mechanisms resulting in sub-epithelial fibrosis and effective therapeutic interventions remains limited. Utilizing a mouse model of chronic challenge to clinically relevant house dust mite, we demonstrate a central role of SP-D in the development of sub-epithelial fibrosis. Our new findings support that SP-D regulates the number of tissue eosinophils and the level of eosinophil-derived TGF-β1 and IL-13. Together our findings provide novel evidence supporting that functional SP-D can protect allergic airways from the development of sub-epithelial fibrosis.

TGF-β is known as a key cytokine of collagen production in fibrotic disease including airway remodeling in asthma [16,21]. TGF-β can induce differentiation of fibroblasts to myofibroblasts, which can contribute to collagen deposition [28] and production of growth factors [29,30]. Previous reports demonstrated anti TGF-β1 or smad3 neutralizing antibody treatment reduced airway remodeling in OVA chronic exposure model [31,32]. In our findings, TGF-β1 production was increased in SP-D−/− mice, contributing to enhance sub-epithelial fibrosis. Interestingly, SP-D can bind to allergens including Dp [6]. Therefore, if functional SP-D is absent, unbound Dp antigen may be a trigger that leads to enhanced TGF-β1production and sub-epithelial fibrosis as observed in SP-D−/− mice.

Previous work has suggested that sub-epithelial fibrosis after chronic challenge to house dust mite antigen was independent of either eosinophils or TGF-β1 [33,34]. In that context, our observation that anti-TGF-β1 antibody treatment reduced sub-epithelial fibrosis and collagen production in the Dp-challenged SP-D deficient mice was quite unexpected. One explanation is that the previous studies used mice that were sufficient in SP-D and the involvement of eosinophils and/or TGF-β1 may not be appreciated until SP-D is absent or dysfunctional.

Alternatively, IL-13 is recognized as a Th2 cytokine that can contribute to sub-epithelial fibrosis and a pro-fibrotic cytokine in lung diseases [35]. IL-13 depletion can reduce sub-epithelial fibrosis and epithelial hypertrophy in chronic asthma model [36,37]. Fattouh *et al.* demonstrated that IL-13 was important for airway fibrosis independent of TGF-β signaling in Th2 associated disease [34,38]. Our findings demonstrated that IL-13 production was increased in Dp-challenged SPD−/− mice and SP-D rescue decrease these responses similar to previous observations [34]. In addition, our findings demonstrate that anti-TGF-β1 antibody treatment decreased IL-13 production in lung homogenate in SPD−/− mice (Figure 9), which suggests a potential synergistic role between TGF-β1 and IL-13 in airway remodeling when SP-D is absent. A previous study found that administration of a soluble TGF-β receptor-Fc molecule ameliorated IL-13-induced fibrosis [39], supporting this paradigm. Recent reports also showed that inhibition of TGF-β1/smad3 signaling can lead to decrease IL-13 production in lung diseases [40,41]. Similar mechanism seems to occur in the lung of SP-D−/− mice, which warrant further study.

In either chronic antigen exposure asthma model or gene-modified model, both peribronchial fibrosis and TGF-β production were related to eosinophils [26,27]. In airways of asthmatic patients, 75-80% of TGF-β1 mRNA expression positive cells were eosinophils [42,43]. On the other hand, it is known that bronchial epithelial cells were also source of TGF-β1 in asthma [44]. In OVA challenged model, bronchial epithelium-derived TGF-β1 was enhanced sub-epithelial fibrosis [31]. Therefore, we examined what type of cells that are a potential source of TGF-β1 and as target cells by SP-D in this model. In the present findings, TGF-β1 expressing eosinophils were increased in Dp-challenged SP-D−/− mice. Moreover, we identified that SP-D directly suppress the production of eosinophil derived TGF-β. To our knowledge, this is the first report to demonstrate a direct function of SP-D on eosinophils response to antigen. In contrast, our findings showed that TGF-β1 expression in bronchial epithelial cells of SP-D−/− mice were not significant difference among the 3 groups unlike TGF expression in eosinopils (Figure 7 and Table 1). Based on these results, our findings suggest that a target of SP-D in Dp-induced sub-epithelial fibrosis may be the activated eosinophils that produce TGF-β1. In addition to regulation of eosinophil-derived TGF-β, SP-D also attenuated IL-13 production from Dp stimulated eosinophils. Since we did not identify an increase Th2 cells in the lung tissue from SP-D−/− mice, our findings suggest that eosinophils may be also an important source of IL-13 as reported in other model systems [45]. Taken together, our findings support that functional SP-D regulates both the tissue infiltration and function of eosinophils, resulting in protection of the airways against development of sub-epithelial fibrosis. Our findings extend the functional role of SP-D in allergic airways disease beyond regulation of eotaxin-triggered chemotaxis and degranulation of eosinophils [46], induction of apoptosis in eosinophils, and enhanced uptake of eosinophils by macrophages [47].

The molecular mechanism that SP-D inhibited TGF-β1 and IL-13 production by Dp-activated eosinophils also remains unknown. Previous reports have shown that interstitial eosinophils express high levels of signal regulatory protein (SIRP)-α, an inhibitory receptor of SP-D, and that cross-linking of SIRP-α on the surface of eosinophils significantly reduced the amount of eosinophil peroxidase released during stimulation with a calcium ionophore [48]. In macrophages under normal condition, SP-D binds SIRP-α, leading to inhibit p38 activation, which induces cytokine production via Src homology region 2 domain-containing phosphatase (SHP)-1 [49,50]. It remains unknown whether similar events occur in eosinophils during chronic allergic inflammatory conditions. Since Toll-like receptor (TLR) 4 is candidate of receptor of Dp [51], it is possible that SP-D bind Dp directly in order to block Dp binding to TLR4. Alternatively, SP-D may also interfere with signaling by binding directly to TLR4. Understanding the molecular mechanisms that SP-D regulates eosinophil function will be the focus of future investigations.

In conclusion, we identify that SP-D regulates eosinophil production of both IL-13 and TGF-β after stimulation with Dp, mitigating sub-epithelial fibrosis which is an important component of airway remodeling in chronic allergic airways disease. Appreciation of the functional role of SP-D during allergic airways disease is high clinical significance since a better understanding of how to attenuate the severity of sub-epithelial fibrosis could lead to better treatment options.

## Additional file

Additional file 1:
**Surfactant Protein D Attenuates Sub-epithelial Fibrosis through Regulation of Eosinophil-derived TGF-β in Chronic Murine Model of Asthma.**
