# Supplementary material for: Surfactant protein D attenuates sub-epithelial fibrosis in allergic airways disease through TGF-β
Source: Respir Res. 2014 Nov 29;15(1):143. doi: 10.1186/s12931-014-0143-9 (PMC4262976; doi:10.1186/s12931-014-0143-9)
Supplement: Additional file 1: — Surfactant Protein D Attenuates Sub-epithelial Fibrosis through Regulation of Eosinophil-derived TGF-β in Chronic Murine Model of Asthma. [file 12931_2014_143_MOESM1_ESM.docx]

**Online Supplement**

**Surfactant Protein D Attenuates Sub-epithelial Fibrosis through Regulation of Eosinophil-derived TGF-β in Chronic Murine Model of Asthma**

Hirohisa Ogawa^1,2,4^, Julie G Ledford^1,2^, Sambbudho Mukherjee^1, 2^, Yoshinori Aono^3^, Yasuhiko Nishioka^3^,　James J. Lee^5^, Keisuke Izumi^4^, John W. Hollingsworth^2,6^

^1^Departments of Cell Biology, Duke University Medical Center, Durham, North Carolina, ^2^Department of Medicine, Duke University Medical Center, Durham, North Carolina,

^3^Department of Respiratory Medicine and Rheumatology, Institute of Health Bioscience, University of Tokushima Graduate School, Tokushima, Japan,

^4^Department of Molecular and Environmental Pathology, Institute of Health Bioscience, University of Tokushima Graduate School, Tokushima, Japan,

^5^Department of Biochemistry and Molecular Biology, Division of Pulmonary Medicine, Mayo Clinic Arizona, Scottsdale, Arizona

^6^Department of Immunology, Duke University Medical Center, Durham, North Carolina

**Materials and Methods**

**Antigen preparation**

House-dust mite antigen (Dermatophagoides pteronyssinus, Dp) was purchased from Cosmobio Ltd (Tokyo, Japan). Endotoxin levels were reduced to < 0.02 EU/mg using endotoxin removal solution (Sigma-Aldrich, Japan).

**Animal protocol**

All mouse studies were conducted in strict accordance with the recommendations of Guide for the Care and Use of Laboratory Animals of the National Institutes of Health. Our protocols were approved by the Institutional Animal Care and Use Committee (IACUC) of Duke University. All surgery was performed while mice were anesthetized with ketamine (50 mg/kg) and xylazine (5 mg/kg), and efforts were made to minimize animal suffering.

SP-D knockout (SP-D-/-) mice (C57BL/6 background) and IL-5 transgenic mice(C57BL/6 background) were generated as previously described[[1](#_ENREF_1), [2](#_ENREF_2)]. Wild type (Wt) C57BL/6 mice were purchased from The Jackson Laboratory and bred in-house with controled environmental conditions. All mice (6-10 weeks old) were sensitized on days 0 and 7 by intraperitoneal injections of 10 µg of Dp (10μl of 1mg/ml of Dp) dissolved in 500 µl of saline and mixed with 1 mg of alum. Mice were anesthetized using intraperitoneal injections. While under anesthesia, Dp-sensitized mice were challenged intranasally with 10 μg (10 μl) of Dp in 70 μl of PBS every other day, 3 days per week, from day 14 to day 39. Mice were sacrificed on day 42 (Figure 1). Each experiment 3-5 mice/group and these experiments were repeated 2-3times.

**Isolation of recombinant SP-D**

Recombinant SP-D was isolated from Chinese hamster ovary cells that expressed a clone of full-length rat SP-D protein and purified using maltose affinity chromatography as described previously [[3](#_ENREF_3)]. SP-D protein was stored at 4°C in 5 mM Tris buffer (pH 7.8), that contained 2 mM EDTA. To confirm that used SP-D was functionally clean, heat inactivated SP-D were prepared. For heat inactivation, SP-D was boiled at 100°C for 10min [[4](#_ENREF_4)].

**Exogenous SP-D administration *in vivo***

Recombinant SP-D (3 μg in 50 μl of PBS) or 50 μl of PBS as control was administrated intratracheally into a Dp-sentitized SP-D-/- mouse twice a week from days 14 to 38 to determine if exogenous SP-D could reduce Dp-induced subepithelial fibrosis on day42 (Figure 1). Briefly, an anesthetized mouse was suspended by its upper incisors using a wire attached to an inclined board. Its tongue was gently extended and PBS with or without SP-D was pipetted into its mouth. Briefly occluding its nose forced the mouse to inhale through its mouth, thereby aspirating the solution into its respiratory tract in one or two breaths. The mouse was then removed from the board and closely observed until it fully recovered from anesthesia.

**Anti-TGF-β1 antibody administration i*n vivo***

Anti-TGF-β1 and IgG isotype antibodies were purchased from R&D Systems (Minneapolis, MN, 0.5mg/ vial) and were dissolved in 1ml of PBS. At 30 minutes before a Dp challenge, 1.0mg/kg of both antibodies were administered intraperitoneally to a Dp challenged SP-D-/- mouse twice weekly from days 14 to 37 (Figure 1).

**Bronchoalveolar lavage**

Bronchoalveolar lavage (BAL) was performed at the time of necropsy. A mouse trachea was cannulated and the lungs were lavaged 2 times with 0.6 ml of PBS. Total BAL cells were collected by centrifugation, and viable cells were counted using a hemocytometer. Cytospin preparations of BAL cells were stained with Hemacolor (EMD Chemicals, Gibbstown, NJ) and used for differential cell counts. The supernatant of recovered BAL fluid was stored at -20ºC for cytokine measurement.

**Homogenization of whole lungs**

After BAL samples collected, the right lungs were quickly frozen in liquid nitrogen. Frozen tissue samples were homogenized in lysis buffer (Cell Signaling Technology, Inc. Danvers, MA) that contained 1mM phenylmethanesulfonyl fluoride (PMSF, Sigma-Aldrich) using a Savant FastPrep FP120 Homogenizer (Thermo Scientific, Waltham, MA). Homogenate samples were centrifuged at 15000 rpm for 20 min, and the supernatants were stored at -80°C for subsequent analysis.

**Histopathology**

Lungs were fixed in 10% buffered formalin and embedded in paraffin. Tissue sections including sequential sections (3 μm thick), were prepared using a microtome. Gomori’s-trichrome staining was done using a commercial kit (Richard Allen Scientific, Kalamazoo, MI). Eosinophils in one sequential section were identified using Luna modified staining. Briefly, a section was stained with 1% Biebrich Scarlet solution for 10min followed by counter staining with haematoxylin. To assess any changes in fibrosis (collagen and elastin), images of the lung were acquired with a Nikon Eclipse 50i microscope at×10 (aperture 0.3) or ×40 (aperture 0.75) at room temperature by digital photography using a Nikon Infinity 2 camera. Infinity Capture software was used for image acquisition.

**Immunohistochemistry**

Immunohistochemistry (IHC) for another sequential section was done using anti-mouse TGF-β1 (Abcam, Cambridge, UK) and anti-IL-13 (Abcam) antibodies according to the manufacturer’s instructions. For TGF-β1staining, sections were first incubated with 100mg/ml of a protein kinase solution at 37 °C for 30 min, and after which the anti-TGF-β1 antibody was applied at 4°C overnight. Then, IHC staining was done using a Vectastain Impress kit (Vector Laboratories, Burlingame, CA). For IL-13 staining, sections were first boiled in a citric acid solution for 15min for antigen retrieval, after which the anti-IL-13 antibody was applied at 4°C overnignt. Then IHC staining was done using a CSA II kit (Dako Japan, Tokyo, Japan). Sections were incubated at room temperature for 10 min in PBS that contained 3,3-diaminobenzidine tetrahydrochloride (Vector Laboratory), and were counterstained with hematoxylin.

**Morphological analysis**

Tissue sections were blinded by a researcher not involved in this study. Morphological measurements were made using a Nikon Eclipse 50i microscope with Image J software (National Institute of Health). To assess infiltrating eosinophils and proliferative collagen positive cells in the subepithelium, the numbers of cells were counted and adjusted by the length of the basement membrane of the target bronchus. Then the thickness of subepithelial fibrosis was determined as follows. The area of subepithelial fibrosis (stained blue) around a bronchus was measured and the average thickness was determined from the area of the positive layer divided by the length of the internal circumference of this area. The Mean thickness values were determined for 8–10 bronchi per left lung lobe.

**Total protein and cytokine concentrations**

Protein concentrations were determined by the BCA method (Pierce, Rockford, IL) using bovine serum albumin (BSA) standards. Interleukin (IL)-4, 5, 13, TGF-β1 (R&D Systems, Minneapolis, MN) and interferon (IFN)-γ (eBioscience, San Diego, CA) were measured with commercial ELISA kits according to the manufacturer’s instructions. Cytokine concentrations in a lung homogenate supernatant were normalized by the total protein concentration in the homogenate. The limits of sensitivity for these ELISA kits were: IL-4 (8pg/ml), IL-5 (8pg/ml), IL-13(8pg/ml), TGF-β1 (8pg/ml), and IFN-γ (4pg/ml).

**Collagen assay**

Total lung collagen was determined using a Sircol Collagen Assay kit (Biocolor Ltd., Belfast, United Kingdom) according to the manufacturer’s instructions [[5](#_ENREF_5)]. Briefly, one lung lobe was harvested on day 42 and homogenized in 0.5 M acetic acid (50 volumes relatives to wet lung weight) that contained about 1 mg/ml of pepsin (Sigma) using a Savant FastPrep FP120 Homogenizer (Thermo Scientific, Waltham, MA). Each sample was incubated at room temperature for 24 hours with stirring. After centrifugation, 100 µl of each supernatant was assayed. One milliliter of Sircol dye reagent, which binds to collagen, was added to each sample, and then mixed for 30 minutes. After centrifugation, the pellets were washed with 0.5 M acetic acid and resuspended in 1 ml of the alkali reagent included in the kit and then absorbance was read at 540nm with a spectrophotometer. Collagen standard solutions were utilized to generate a standard curve. Collagens contain about 14% hydroxyproline by weight, and the collagen contents obtained with this method were well correlate with hydroxyproline contents based on the manufacturer’s data. These data were normalized by the total protein of homogenized lung.

**Eosinophil purification and in vitro experiment**

Eosinophils were purified from the blood of an IL-5 transgenic mouse as described previously [[6](#_ENREF_6)]

Briefly, blood was collected during necropsy from an IL-5 transgenic mouse via cardiac puncture into 0.5 ml EDTA tubes (Sarstedt). RBCs were lysed and cells were resuspended in 0.1% BSA in PBS. Biotin labeled antibodies (1 μl per 10^7^ cells) against B220 (CD45R) and Thy 1.2 (CD90.2) were incubated with cells on ice for 15 minutes. Cells were then resuspended in 0.1% BSA and Dynabeads were added (1 μg/10^6^ target cells up to 50 μl) and incubated at 4°C for 30 minutes with rotation every 5 minutes. Samples were then placed into the magnetic apparatus with 4 ml of additional buffer for 5 minute increments to deplete magnetically labeled cells. This yielded a population of eosinophils of> 95% purity as confirmed by H&E staining.

For *in vitro* experiments, eosinophils (4x10^5^/well) were pre-incubated in a 48 well plate with 5% FBS/ DMEM in either the presence or absence of different concentrations (2 μg/ml to 5μg/ml) of SP-D or heat inactivated SP-D (HiSP-D) for 1hr. Then eosinophils were stimulated with different concentrations (50μg/ml to 100μg/ml) of Dp solution for 24hrs. After 24hr, supernatants were collected and stored at -80°C until analyzed.

**Lung cells / lymphocyte isolation and flow cytometry for lymphocyte intracellular staining.**

After a mouse was sacrificed, its lungs were harvested, minced with razor blades, and treated with collagenase A (Roche Applied Science, Indianapolis, IN) and DNAse I (Worthington, Lakewood, NJ) for 30 min. Cells were filtered through a 40-μm nylon strainer to obtain a single-cell suspension. To isolate lymphocytes, a cell suspension was subjected to density gradient centrifugation using Ficoll-Hypaque 1083 (Sigma-Aldrich). RBC lysing solution (BioLegend) was used to remove any residual RBCs. Multiple panels for multicolor T cell surface staining were set up using PerCP-Cy5.5 labeled anti-CD3 (eBioscience or BioLegend),PE-Texas Red labeled anti-CD4 (Life technology) or AF647 labeled anti-CD4 (BioLegend) to identify specific cell populations. For intracellular staining using FITC labeled anti IL-4 (e-bioscience) and PECy7- labeled anti IFN-γ (e-bioscience), cells were washed well after surface staining, fixed in 10% neutral buffered formalin, and permeabilized with 0.3% saponin. Cells were then stained with anti IL-4 and anti IFN-γat 4ºC overnight. Stained cells were analyzed using a BD LSRII or BD FACS Canto II (San Diego, CA) and FloJo software (Treestar, Inc. Ashland, OR). Lymphocytes were examined for their expression of both CD3 and CD4 using contour plots by quadrant analysis. IL-4/IFN-γ positive cells were then analyzed among CD3/CD4+ T cells.

**Statistical Analysis**

Results were statistically compared by student T-test, unless otherwise noted. Statistical analysis was done using GraphPad Prism, version 5.0 (GraphPad Software, Inc., San Diego, CA). P values of <0.05 were considered significance.

**REFERENCE**

1. Lee NA, McGarry MP, Larson KA, Horton MA, Kristensen AB, Lee JJ, Expression of IL-5 in thymocytes/T cells leads to the development of a massive eosinophilia, extramedullary eosinophilopoiesis, and unique histopathologies*.* Journal of immunology 1997;158: 1332-44.

2. Wert SE, Yoshida M, LeVine AM, Ikegami M, Jones T, Ross GF, Fisher JH, Korfhagen TR, Whitsett JA, Increased metalloproteinase activity, oxidant production, and emphysema in surfactant protein D gene-inactivated mice*.* Proceedings of the National Academy of Sciences of the United States of America 2000;97: 5972-77.

3. Dong Q, Wright JR, Degradation of surfactant protein D by alveolar macrophages*.* American Journal of Physiology 1998;274: L97-105.

4. Pasula R, Wright JR, Kachel DL, Martin WJ, 2nd, Surfactant protein A suppresses reactive nitrogen intermediates by alveolar macrophages in response to Mycobacterium tuberculosis*.* The Journal of clinical investigation 1999;103: 483-90.

5. Ogawa H, Azuma M, Muto S, Nishioka Y, Honjo A, Tezuka T, Uehara H, Izumi K, Itai A, Sone S, I kappa B kinase beta inhibitor IMD-0354 suppresses airway remodelling in a Dermatophagoides pteronyssinus-sensitized mouse model of chronic asthma*.* Clinical and Experimental Allergy 2011;41: 104-15.

6. Ledford JG, Mukherjee S, Kislan MM, Nugent JL, Hollingsworth JW, Wright JR, Surfactant Protein-A Suppresses Eosinophil-Mediated Killing of Mycoplasma pneumoniae in Allergic Lungs*.* Plos One 2012;7.
